# Dynamic and Baseline Multi-Task Learning for Predicting Substance Use Initiation in the ABCD Study

**DOI:** 10.64898/2026.04.10.26350655

**Published:** 2026-04-13

**Authors:** Mengman Wei, Hanwen Zhang, Qian Peng

**Affiliations:** 1 Department of Neuroscience, The Scripps Research Institute, 10550 N Torrey Pines Rd, La Jolla, 92037, CA, U.S.; 2 University of California, San Diego, 9500 Gilman Dr, La Jolla, CA, 92093, USA

**Keywords:** Multi-Task Learning, Substance Use Initiation, Longitudinal Analysis, ABCD

## Abstract

**Background:**

Early initiation of substance use is linked to later adverse outcomes, and risk factors come from multiple domains and are shared across substances. In our previous work, traditional time-to-event Cox models identified individual risk factors, but these models are not designed to jointly model multiple outcomes or capture complex non-linear relationships. Multi-task learning (MTL) can leverage shared structure across related outcomes to improve prediction and distinguish common versus substance-specific predictors. However, most MTL studies rely on baseline features and focus on single outcomes, which limits their ability to capture shared risk and temporal changes. Substance use initiation is a time-dependent process that unfolds during development and reflects changing exposures over time. Baseline-only models cannot capture these changes or represent risk dynamics. Discrete-time modeling provides a practical approach by estimating interval-level initiation risk and combining it into cumulative risk at the subject level. By integrating multi-task learning with dynamic modeling, it is possible to share information across outcomes while capturing how risk evolves over time, which may improve prediction performance.

**Methods:**

Using the Adolescent Brain Cognitive Development (ABCD) Study^®^ (release 5.1), we developed two complementary multi-task learning (MTL) frameworks to predict initiation of alcohol, nicotine, cannabis, and any substance use. A baseline MTL model predicted fixed-horizon (48-month) initiation using one record per participant, while a dynamic discrete-time MTL model incorporated longitudinal interval data to model time-varying risk. Both models used multi-domain environmental exposures, core covariates, and polygenic risk scores (PRS). Performance was evaluated on a held-out test set using AUROC, PR-AUC, and calibration metrics, and compared with single-task logistic regression (LR). Feature importance was assessed using permutation importance and compared with Cox proportional hazards models.

**Results:**

MTL showed comparable or improved performance relative to LR, with larger gains for low-prevalence outcomes (cannabis and nicotine). Incorporating longitudinal information led to consistent improvements across all outcomes. Dynamic models increased AUROC by +0.044 to +0.062 for MTL and +0.050 to +0.084 for LR, indicating that temporal information was the primary driver of performance gains. Feature importance analyses showed modest overlap across methods, with higher agreement between dynamic MTL and Cox models than static MTL. A small set of features, including externalizing behavior, parental monitoring, and developmental factors, were consistently identified across all approaches.

**Conclusions:**

Dynamic multi-task learning improves the prediction of substance use initiation by leveraging longitudinal structure and shared information across outcomes. While MTL provides additional gains, incorporating time-varying information is the dominant factor for improving performance. Combining baseline and dynamic frameworks offers a comprehensive strategy for identifying robust risk factors and modeling adolescent substance use initiation.

## Introduction

Substance use often begins during adolescence, and earlier initiation is associated with increased risk of later substance use disorders and adverse psychosocial outcomes [1, 2, 3, 4]. Predicting initiation is challenging because risk arises from multiple interacting domains, including individual behavior, mental health, family environment, peers, neighborhood context, and broader sociodemographic factors [5, 6, 7]. Many of these factors are shared across substances, while some are substance-specific [8, 9, 10]. Most prior modeling approaches treat each substance outcome separately, which may fail to capture shared structure across related behaviors. Multi-task learning (MTL) provides a framework to jointly model correlated outcomes by learning shared representations while allowing task-specific prediction components [11, 12]. This approach can improve performance in settings with limited positive cases and help distinguish predictors that generalize across outcomes from those that are outcome-specific.

However, many prediction studies rely only on baseline features and do not account for how risk changes over time. Since substance use initiation unfolds longitudinally, models that incorporate temporal structure may better reflect real-world risk processes.

**Figure 1:**
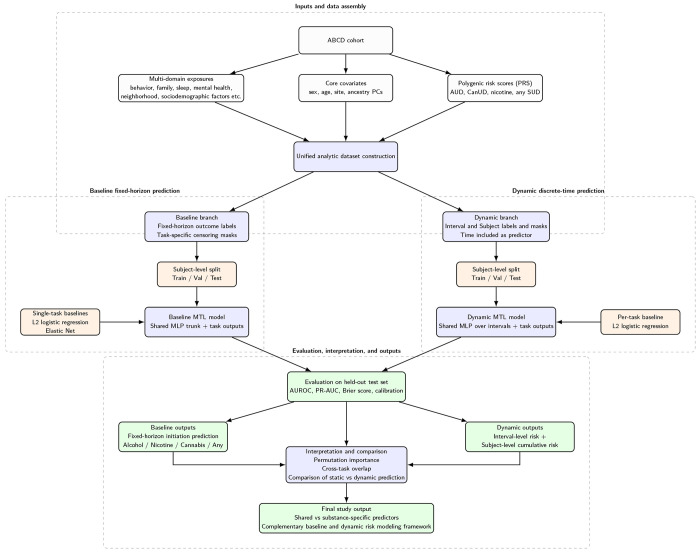
Overview of the multi-task learning pipeline for predicting substance use initiation in the ABCD Study. Multi-domain exposures, core covariates, and polygenic risk scores were assembled into a unified analytic dataset. Two complementary modeling branches were implemented: (1) a baseline fixed-horizon multi-task model using one baseline record per participant and (2) a dynamic discrete-time multi-task model using intervalized longitudinal observations. Both frameworks predicted initiation for alcohol, nicotine, cannabis, and any substance use, and were compared with logistic-regression baselines. Model performance was evaluated on held-out test data using discrimination and calibration metrics, and predictors were summarized using feature-importance and overlap analyses.

In this study, we propose an MTL pipeline (Figure 1) for predicting initiation of alcohol, nicotine, cannabis, and any substance use in the ABCD [13, 14]cohort using multi-domain exposures, core covariates, and polygenic risk scores (PRS) [15]. We evaluate predictive performance using discrimination and calibration metrics on a held-out test set, compare against interpretable single-task performance, and assess feature importance and cross-outcome overlap [16, 17, 18, 19, 20, 21].

To address both shared structure and temporal dynamics, we integrate two complementary approaches: a baseline fixed-horizon MTL model and a dynamic discrete-time MTL model. The baseline model summarizes risk within a fixed prediction window, while the dynamic model represents time-varying risk across longitudinal intervals. This combined framework allows direct comparison between static and dynamic prediction strategies.

## Method

### Study Cohort

We used data from the ABCD Study^®^ release 5.1, a large U.S. multisite prospective longitudinal cohort that enrolled approximately 11,880 children aged 9-10 years across 21 research sites and follows them into adolescence and early adulthood. The study includes harmonized assessments spanning neuroimaging, cognition, mental and physical health, substance use, and environmental and sociodemographic measures.

In the analytic cohort (*N* = 11,868; mean baseline age 9.91 ± 0.62 years), substance-use initiation events were observed by the end of follow-up for alcohol (4,330/11,868; 36.5%), nicotine (646/11,868; 5.44%), cannabis (406/11,868; 3.42%), and any substance (4,706/11,868; 39.7%). Among participants with events, the mean (SD) time to initiation (in months from baseline) was 20.83 (1.82) for alcohol (median 20.75; IQR 19.42–21.83), 22.65 (1.93) for nicotine (median 22.83; IQR 21.33–24.17), 23.30 (1.70) for cannabis (median 23.42; IQR 22.19–24.65), and 20.93 (1.85) for any substance (median 20.92; IQR 19.50–21.92).

Event rates were slightly higher in males than females for alcohol (39.6% vs. 36.7%) and any substance (43.1% vs. 39.7%). Nicotine initiation rates were comparable between sexes (5.29% in males vs. 6.02% in females), and cannabis initiation rates were similar (3.76% vs. 3.51%). Participants with unknown sex exhibited lower event rates across all outcomes (alcohol 26.6%, nicotine 4.36%, cannabis 2.18%, any substance 29.4%).

Across race/ethnicity groups, alcohol initiation rates were highest among White participants (42.7%; *n* = 6,173), followed by Other (37.3%; *n* = 1,248), Hispanic (32.6%; *n* = 2,410), Asian (33.3%; *n* = 252), and Black participants (20.1%; *n* = 1,784). A similar pattern was observed for any-substance initiation, with rates of 44.8% (White), 40.9% (Other), 36.9% (Hispanic), 34.1% (Asian), and 25.3% (Black). Nicotine and cannabis initiation were generally lowest among Asian participants (0.79% and 0.40%, respectively). Additional details are provided in our previous work [22].

### Train/validation/test splitting (subject-level)

We created a subject-level split with zero IID overlap across train/validation/test sets using a single random permutation with seed and proportions 70/15/15 (train/val/test). The split universe was defined by unique participants present in the covariate table (IID). Split manifests were saved for reproducibility.

### Baseline multi-task model

#### Baseline feature construction

We derived baseline features from the time-varying covariate table by retaining a single record per participant, defined as the earliest available visit (i.e., the minimum value of time months).

Baseline features included three components: (1) numeric exposure variables measured at baseline; (2) core covariates, including sex, age at baseline, study site, and genetic ancestry principal components (PC1–PC20); and (3) polygenic risk scores (PRS) for alcohol use disorder, cannabis use disorder, nicotine use disorder, and any substance use disorder, merged by participant identifier (IID) when available.

Missing values were handled by converting variables to numeric where possible, followed by median imputation during model training. Any remaining missing values were set to 0.

#### Outcome definition: baseline and horizon classification with censoring masks

For each substance outcome (alcohol, nicotine, cannabis, and any substance use), we used ABCD time-to-event data that include an event indicator and an event or censoring time. Because times are often recorded as absolute age in months, we converted them to follow-up time since baseline by subtracting each participant’s baseline age (in months).

We defined a binary initiation label using a fixed prediction horizon *H* months (primary *H* = 48). Participants were labeled as positive if initiation occurred before *H* . They were labeled as negative if they remained initiation-free for at least H months (i.e., initiation occurred after *H*, or no initiation with follow-up ≥ *H*).

If a participant was censored before *H* (no event and follow-up < *H*) or had missing event/time information, the label was treated as unobserved and excluded for that outcome. Participants were retained if they had at least one observed outcome label across all tasks.

#### Multi-task model architecture, loss, hyperparameter optimization, and early stopping

We trained a multi-task neural network with a shared multilayer perceptron (MLP) trunk and task-specific outputs for alcohol, nicotine, cannabis, and any substance use, following standard multi-task learning practice [11].

##### Notation.

Let t ∈{1,…,T} index tasks and i∈{1,…,N} index individuals. We define:

- $m_{it}\in \{0,1\}$: label-observed mask (1 if the label exists for individual i in task t),
- *αt* : task weight,
- pos_weight_*t*_ : positive-class weight for class imbalance,
- *z_it_* : model logit output,
- *y_it_* : observed binary label.

##### Model architecture.

The shared trunk used ReLU activations and dropout for regularization [23]. We evaluated two output designs: (i) a single joint output layer producing logits for all tasks, and (ii) task-specific adapter modules with separate output heads to allow limited task customization on top of shared representations [12].

##### Loss function.

Model training minimized masked binary cross-entropy with logits:

(1) $$ℒ=\overset{T}{\underset{t=1}{\sum }}\alpha_{t}\cdot \frac{1}{\sum_{i=1}^{N}m_{\mathrm{it}}}\overset{N}{\underset{i=1}{\sum }}m_{\mathrm{it}}\cdot \text{BCEWithLogits}\left(z_{\mathrm{it}},y_{\mathrm{it}};{\text{pos weight}}_{t}\right).$$

This formulation ensures that each individual contributes to the loss only for tasks with observed labels. To address class imbalance, we used task weights *α_t_* and task-specific positive-class weights pos weight_*t*_ [24, 25].

##### Hyperparameter optimization and training.

We tuned model architecture and optimization parameters, including hidden layer sizes, dropout rate, learning rate, weight decay, batch size, and loss weights, using Optuna with the Tree-structured Parzen Estimator (TPE) sampler and optional median pruning [26].

Early stopping was applied based on a validation objective combining performance across tasks (AUROC and PR-AUC), with training stopped when improvement plateaued over a fixed patience window [27].

#### Baseline models (single-task)

As interpretable single-task baselines, we fit a separate regularized logistic regression model for each outcome (alcohol, nicotine, cannabis, and any substance use), using only participants with an observed label for the corresponding outcome.

We used L2-regularized logistic regression (ridge) to improve stability in the presence of correlated predictors [28, 29, 30].

### Dynamic discrete-time multi-task model

#### Dynamic multi-task neural network.

We trained a multi-task neural network to predict initiation risk for all outcomes at the interval level. Input features included all numeric variables, excluding identifiers and bookkeeping columns. Time variables were included as numeric predictors.

All features were coerced to numeric format. Columns that became entirely missing after coercion were removed. Missing feature values were imputed using the median, and any remaining missing values were set to 0. We standardized all features using a StandardScaler (scikit-learn) fit on the training split only, and applied the same transformation to the validation and test splits [31].

The neural network consisted of a shared multilayer perceptron (MLP) trunk with two hidden layers (256 and 128 units), ReLU activations, and dropout (rate = 0.2) for regularization [32]. A single linear output layer produced one logit per task for each interval, and predicted probabilities were obtained using the sigmoid function.

##### Loss function and training.

Let *z_it_* denote the model logit for interval *i* and task *t*, *y_it_* the binary label, and $m_{it}\in \{0,1\}$ the label mask (1 indicates an observed label). We minimized masked binary cross-entropy with logits:

(2) $$ℒ=\frac{\sum_{i,t}m_{\mathrm{it}}\cdot \text{BCEWithLogits}\left(z_{\mathrm{it}},y_{\mathrm{it}}\right)}{\sum_{i,t}m_{\mathrm{it}}}.$$

This formulation ensures that each interval contributes to the loss only for tasks with observed labels.

We used the Adam optimizer with weight decay for training. Models were trained for up to a fixed maximum number of epochs, with early stopping based on masked validation loss using a fixed patience. Gradient clipping was applied to improve training stability. The final model was selected based on the lowest validation loss and evaluated on the held-out test split.

#### Evaluation and feature importance

##### Interval-level evaluation.

On the test split, interval-level metrics were computed separately for each task using only intervals with observed labels (*m_it_* = 1). We reported the area under the receiver operating characteristic curve (AUROC) and area under the precision–recall curve (PR-AUC). We also computed the Brier score and classification accuracy using a probability threshold of 0.5.

##### Subject-level cumulative risk.

To obtain subject-level predictions, we aggregated interval-level probabilities across all observed intervals for each subject in the test split (*m_it_* = 1). For a subject with interval probabilities {*p_k_*}, cumulative risk was defined as:

(3) $$\text{CumRisk}=1-\prod_{k}\;\left(1-p_{k}\right).$$

Subject-level ground truth was defined as whether the subject had at least one interval with *y_it_* = 1 for the corresponding task among observed intervals. We evaluated subject-level performance using AUROC and PR-AUC.

To quantify uncertainty, we estimated 95% confidence intervals using bootstrap resampling over subjects (sampling subject IDs with replacement, repeated a fixed number of times), and computed the 2.5th and 97.5th percentiles of the bootstrap distribution.

##### Baseline model (logistic regression).

As a baseline, we trained a separate L2-regularized logistic regression model for each task. For each outcome, the model was fit on training intervals with observed labels (*m_it_* = 1) and evaluated on test intervals with observed labels. We reported interval-level AUROC and PR-AUC for the baseline.

##### Permutation feature importance.

We computed permutation feature importance on the test split as the decrease in interval-level AUROC after permuting one feature at a time. Importance was calculated separately for each task and averaged across a small number of repeats. To control computational cost, the number of evaluated features was limited to a predefined maximum.

### Evaluation and comparison with survival and causal-style estimates

On the held-out test set, we evaluated model discrimination using the area under the receiver operating characteristic curve (AUROC) and the area under the precision–recall curve (PR-AUC), with PR-AUC emphasized due to its greater informativeness under class imbalance [33].

Calibration was assessed using the Brier score, expected calibration error (ECE), and calibration curves, to evaluate the agreement between predicted probabilities and observed event rates [34].

To characterize feature importance, we computed permutation importance as the decrease in test AUROC after randomly permuting each feature, thereby breaking its association with the outcome [35].

To place predictive importance in an inferential context, we compared top-ranked machine learning features for each outcome with results from time-varying Cox models, including hazard ratios and multiple-testing–adjusted significance, and, where available, marginal structural model (MSM) estimates. We examined the extent to which features identified as important for prediction also showed statistically supported associations in survival and causal analyses [22, 36, 37].

## Results

### Cohort construction and data characteristics

Cohort construction and baseline feature harmonization followed our prior work and are described in detail elsewhere [22]. Briefly, we constructed a baseline dataset from the ABCD time-varying covariate table and generated 48-month horizon initiation labels for four outcomes (alcohol, nicotine, cannabis, and any substance use).

Among 11,868 participants available for splitting, applying the horizon-based label-masking filter (requiring ≥ 1 observed outcome label) yielded a final analytic cohort of 11,187 participants (94.2% retained). The dataset initially included 107 baseline predictors; during preprocessing, 56 predictors that were entirely missing were removed, resulting in 51 features used for model fitting.

Participants were split at the subject level with no overlap between training, validation, and test sets. In the tuned MTL run, the effective sample sizes were 7,824 (training), 1,678 (validation), and 1,685 (test). Because labels were masked when participants were censored before the 48-month horizon, the number of observed labels differed across outcomes. In the held-out test set (*n* = 1,685), the number of observed labels was 1,680 for alcohol, 1,685 for any substance, 1,634 for cannabis, and 1,638 for nicotine, corresponding to 5, 0, 51, and 47 participants, respectively, with unobserved labels at the 48-month horizon.

Class balance varied substantially across outcomes. In the test set, initiation prevalence among observed labels was 35.9% for alcohol (603/1,680) and 38.4% for any substance (647/1,685), whereas cannabis (37/1,634; 2.26%) and nicotine (67/1,638; 4.09%) were substantially rarer. This imbalance motivated the use of PR-AUC alongside AUROC for evaluation and the application of class- and task-weighted training objectives.

#### Static and dynamic multi-task learning (MTL) versus logistic regression (LR) model performance

We first compared the performance of static MTL and logistic regression (LR) models across four substance initiation outcomes (Table 1). In the static setting, MTL showed comparable or improved discrimination relative to LR. For alcohol initiation, AUROC was similar between models (0.680 vs. 0.682), while MTL achieved higher PR-AUC (+0.025). For any substance initiation, MTL slightly improved AUROC (+0.001) and yielded a notable increase in PR-AUC (+0.030). Larger gains were observed for cannabis and nicotine initiation, where MTL improved AUROC by +0.039 and +0.030, respectively, and substantially increased PR-AUC (+0.050 and +0.039). These results indicate that MTL provides greater benefit for more challenging or lower-prevalence outcomes.

We next evaluated dynamic models that incorporate longitudinal information (Table 2). In this setting, MTL consistently performed as well as or better than LR across most outcomes. For alcohol and any substance initiation, MTL improved AUROC by +0.007 and +0.009, respectively, with corresponding gains in PR-AUC (+0.010 and +0.014). For cannabis initiation, MTL achieved higher AUROC (+0.016) and PR-AUC (+0.026). For nicotine initiation, AUROC was comparable between models (−0.003), but MTL provided a substantial improvement in PR-AUC (+0.042). Overall, dynamic MTL maintained or improved performance relative to LR, particularly in terms of precision–recall performance.

To assess the impact of temporal information, we compared static and dynamic models within each framework (Table 3). Dynamic modeling led to consistent improvements in AUROC across all outcomes for both MTL and LR. For MTL, AUROC increased by +0.044 to +0.062 across tasks, while LR showed gains of +0.050 to +0.084. The largest improvements were observed for cannabis and nicotine initiation, suggesting that temporal patterns play an important role in predicting these outcomes. Notably, the improvement from dynamic modeling exceeded that of switching from LR to MTL in the static setting, indicating that incorporating longitudinal information is the primary driver of performance gains.

Complete results are provided in the Supplementary Material.

Taken together, these results highlight two key findings. First, multi-task learning improves predictive performance over logistic regression, particularly for low-prevalence or complex outcomes. Second, incorporating temporal information through dynamic modeling yields larger performance gains across all outcomes, indicating that longitudinal structure is the dominant factor in improving prediction.

#### Feature importance across MTL models and concordance with Cox models

We compared feature importance derived from static multi-task learning (MTL), dynamic MTL, and Cox proportional hazards models to assess the consistency of identified risk factors across modeling approaches. Overall, agreement between methods was modest, reflecting differences in model objectives and assumptions.

**Figure 2:**
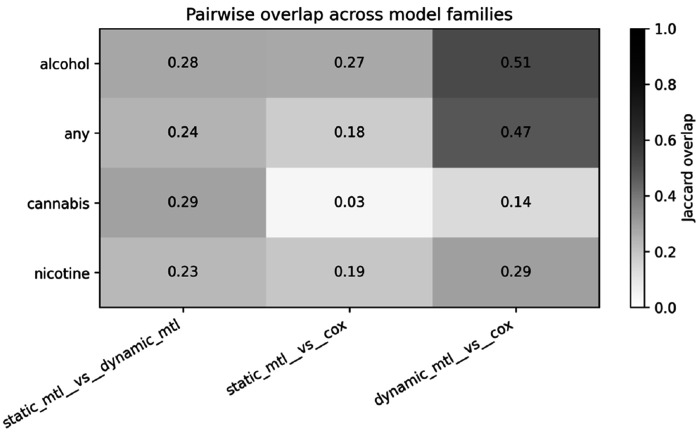
Pairwise feature overlap across modeling frameworks. Heatmap showing Jaccard overlap between static MTL, dynamic MTL, and Cox models across four outcomes (alcohol, any substance, cannabis, and nicotine). Darker colors indicate higher overlap.

Pairwise overlap, quantified using the Jaccard index (Figure 2), showed moderate agreement between static and dynamic MTL models across all outcomes (0.23–0.29). In contrast, agreement between MTL models and Cox models was lower, particularly for cannabis initiation (0.03 for static MTL vs. Cox), indicating that features important for prediction are not always statistically significant in survival models.

Dynamic MTL showed higher agreement with Cox models than static MTL (e.g., alcohol: 0.51 vs. 0.27; any substance: 0.47 vs. 0.18), suggesting that incorporating time-varying information improves alignment with time-to-event modeling.

Although overall overlap was limited, a small subset of features was consistently identified across all three methods. These shared features likely represent stable and robust risk factors, whereas most other features appear to be model-specific.

#### Reproducible shared features across modeling frameworks

**Figure 3:**
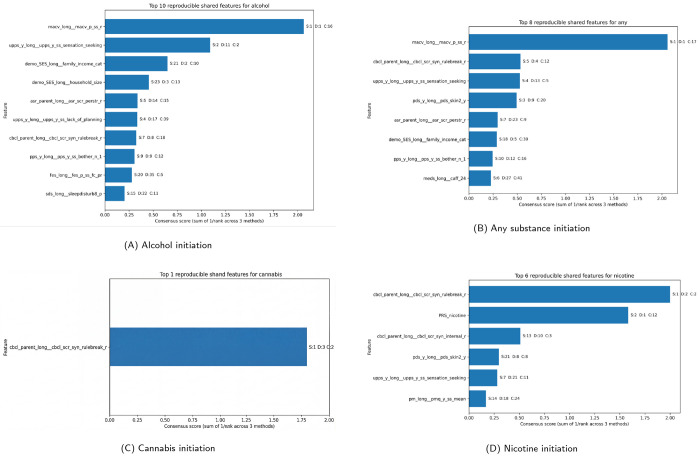
Top reproducible shared features across modeling frameworks. Top consensus-ranked features for each outcome that are identified by all three methods. Bars represent the consensus score (sum of inverse ranks across models). Labels indicate feature ranks in static MTL (S), dynamic MTL (D), and Cox models (C).

We next focused on features that were identified by all three methods (static multi-task learning (MTL), dynamic MTL, and Cox models). These features were ranked using a consensus score that combines rankings across models (Figure 3). This approach prioritizes features that are consistently important rather than those selected by a single method.

##### Alcohol initiation.

For alcohol initiation, several reproducible features were identified. The strongest signal was parental monitoring/child access (MACV), which ranked first in both MTL models and remained highly ranked in the Cox model. Behavioral traits related to impulsivity, including sensation seeking and lack of planning (UPPS), were also consistently identified. Family socioeconomic factors, such as household income and household size, were retained across all methods. Parental stress and rule-breaking behavior (CBCL) were also reproducible predictors, while sleep disturbance and family environment variables showed weaker but consistent signals. Overall, alcohol initiation reflects a combination of behavioral traits, family environment, and socioeconomic context.

##### Any substance initiation.

For any substance initiation, a similar pattern was observed. Parental monitoring (MACV) remained the strongest and most consistent predictor across all models. Behavioral factors, including rule-breaking behavior (CBCL) and sensation seeking (UPPS), were again consistently identified. Developmental measures such as pubertal status (PDS) were also shared features. Environmental variables, including parental stress, family income, and caffeine exposure, were retained across models, although their rankings varied. These results suggest that general substance initiation reflects a combination of behavioral risk, developmental stage, and environmental exposure.

##### Cannabis initiation.

For cannabis initiation, only one feature—rule-breaking behavior (CBCL)—was consistently identified across all three methods. This limited overlap likely reflects the lower prevalence and higher variability of cannabis initiation, as well as reduced statistical power in Cox models. Despite this, the consistent identification of rule-breaking behavior suggests that externalizing behavior is a core risk factor under strict selection criteria.

##### Nicotine initiation.

For nicotine initiation, several reproducible features were identified. Rule-breaking behavior (CBCL) was the most consistent predictor across all methods. Polygenic risk for nicotine use (PRS_nicotine) was also identified, indicating that genetic risk contributes independently to initiation. Internalizing symptoms (CBCL internalizing) were additionally selected, suggesting a role for emotional factors. Developmental measures (PDS) and behavioral traits such as sensation seeking (UPPS) were also included, while family-related variables showed greater variability across models. Overall, nicotine initiation appears to be influenced by genetic risk, behavioral traits, and emotional factors.

##### Overall summary.

Across all outcomes, only a small subset of features was shared across models, while most features were model-specific. The shared features—primarily externalizing behavior, family environment, and developmental factors—likely represent robust and generalizable predictors of substance use initiation.

## Discussion

This study integrates baseline and dynamic multi-task learning (MTL) approaches to model substance use initiation in adolescence. These two frameworks provide complementary views of risk. Baseline models summarize risk within a fixed time window, while dynamic models capture how risk changes over time.

Our results show that the benefit of MTL depends on both outcome prevalence and modeling setting. In the baseline setting, MTL provided modest improvements over logistic regression (LR), with larger gains for low-prevalence outcomes such as cannabis and nicotine initiation. This is consistent with the ability of MTL to share information across related tasks when data are limited. For more common outcomes, such as alcohol and any substance initiation, performance differences between MTL and LR were small, suggesting that well-regularized single-task models already capture much of the available signal.

In contrast, incorporating longitudinal information led to consistent and larger improvements across all outcomes. Dynamic modeling improved AUROC for both MTL and LR, with gains exceeding those observed when switching from LR to MTL in the baseline setting. This indicates that temporal information is the primary driver of performance improvement. Modeling risk as a time-dependent process allows the model to capture evolving exposures and better reflect the underlying developmental process of substance initiation.

The dynamic MTL framework also provides practical advantages. By modeling interval-level risk and aggregating it into subject-level cumulative risk, it produces predictions that are more aligned with real-world risk trajectories. This formulation allows direct interpretation of how risk accumulates over time, which is not possible in baseline-only models.

Feature importance analyses further highlight differences between modeling approaches. Agreement across methods was modest, reflecting differences in model objectives. MTL focuses on predictive performance, while Cox models emphasize statistical association. However, dynamic MTL showed higher agreement with Cox models than static MTL, suggesting that incorporating time-varying information improves alignment with time-to-event analyses. Across all methods, a small set of features—including externalizing behavior, parental monitoring, and developmental factors—were consistently identified, indicating a stable core risk structure.

These findings suggest that MTL should not be viewed as universally superior to simpler models. Instead, its benefits depend on the data structure and prediction goal. When outcomes are imbalanced or related, MTL provides advantages. However, incorporating longitudinal information is more important than model complexity alone.

Overall, this study supports the use of dynamic multi-task learning as a practical extension of traditional models for predicting substance use initiation, particularly when integrating multi-domain and time-varying risk factors.

## Conclusion

This study presents a unified framework combining baseline and dynamic multi-task learning to predict substance use initiation. The results show that incorporating longitudinal information leads to substantial improvements in predictive performance across all outcomes, while multi-task learning provides additional benefits, particularly for low-prevalence outcomes.

Dynamic modeling captures how risk evolves over time and produces subject-level cumulative risk estimates that are more aligned with real-world processes. At the same time, baseline models provide a simpler summary of risk within a fixed horizon. Together, these approaches offer complementary insights.

Across modeling frameworks, a small set of consistent predictors, including behavioral traits, family environment, and developmental factors, emerged as robust risk factors. Most other features were model-specific, highlighting the importance of using multiple approaches to identify stable signals.

Overall, combining dynamic modeling with multi-task learning provides a flexible and effective strategy for predicting substance use initiation and identifying key risk factors in longitudinal data.

## Figures and Tables

**Table 1:** Performance of static multi-task learning (MTL) and logistic regression (LR) models

Task	MTL AUROC	LR AUROC	Δ AUROC	MTL PR-AUC	LR PR-AUC	Δ PR-AUC
Alcohol	0.680	0.682	−0.002	0.532	0.507	+0.025
Any	0.672	0.671	+0.001	0.554	0.524	+0.030
Cannabis	0.741	0.702	+0.039	0.108	0.057	+0.050
Nicotine	0.789	0.759	+0.030	0.177	0.138	+0.039

**Table 2:** Performance of dynamic multi-task learning (MTL) and logistic regression (LR) models

Task	MTL AUROC	LR AUROC	Δ AUROC	MTL PR-AUC	LR PR-AUC	Δ PR-AUC
Alcohol	0.739	0.732	+0.007	0.634	0.624	+0.010
Any	0.734	0.725	+0.009	0.656	0.642	+0.014
Cannabis	0.802	0.786	+0.016	0.191	0.165	+0.026
Nicotine	0.833	0.837	−0.003	0.343	0.301	+0.042

**Table 3:** Comparison of static and dynamic models using AUROC

Task	MTL Static	MTL Dynamic	Δ	LR Static	LR Dynamic	Δ
Alcohol	0.680	0.739	+0.059	0.682	0.732	+0.050
Any	0.672	0.734	+0.062	0.671	0.725	+0.054
Cannabis	0.741	0.802	+0.061	0.702	0.786	+0.084
Nicotine	0.789	0.833	+0.044	0.759	0.837	+0.078

## Data Availability

**Code.** The analysis code and scripts used in this study are publicly available at: https://github.com/mw742/ABCD-MultitasksML and https://github.com/mw742/ABCD-MultitasksML-dynamic. **Data.** This study uses data from the Adolescent Brain Cognitive Development (ABCD) Study (https://abcdstudy.org), available through the NIMH Data Archive (NDA). The ABCD data release used was version 5.1. The study is supported by the National Institutes of Health (NIH) and additional federal partners under multiple award numbers, including U01DA041048 and U01DA050987. A full list of funders is available at https://abcdstudy.org/federal-partners.html.
